# Supplementary material for: Evaluating aerosol and splatter following dental procedures: Addressing new challenges for oral health care and rehabilitation
Source: J Oral Rehabil. 2020 Oct 8;48(1):61–72. doi: 10.1111/joor.13098 (PMC7537197; doi:10.1111/joor.13098)
Supplement: Supplementary file 1 — Fig S1 [file JOOR-48-61-s002.pdf]

Evaluating aerosol and splatter following dental procedures: addressing new challenges for oral healthcare and rehabilitation

Allison JR, Currie CC, Edwards DC, Bowes C, Coulter J, Pickering K, Kozhevnikova E, Durham J, Nile CJ, Jakubovics N, Rostami N, Holliday R

Supplementary Figure 1

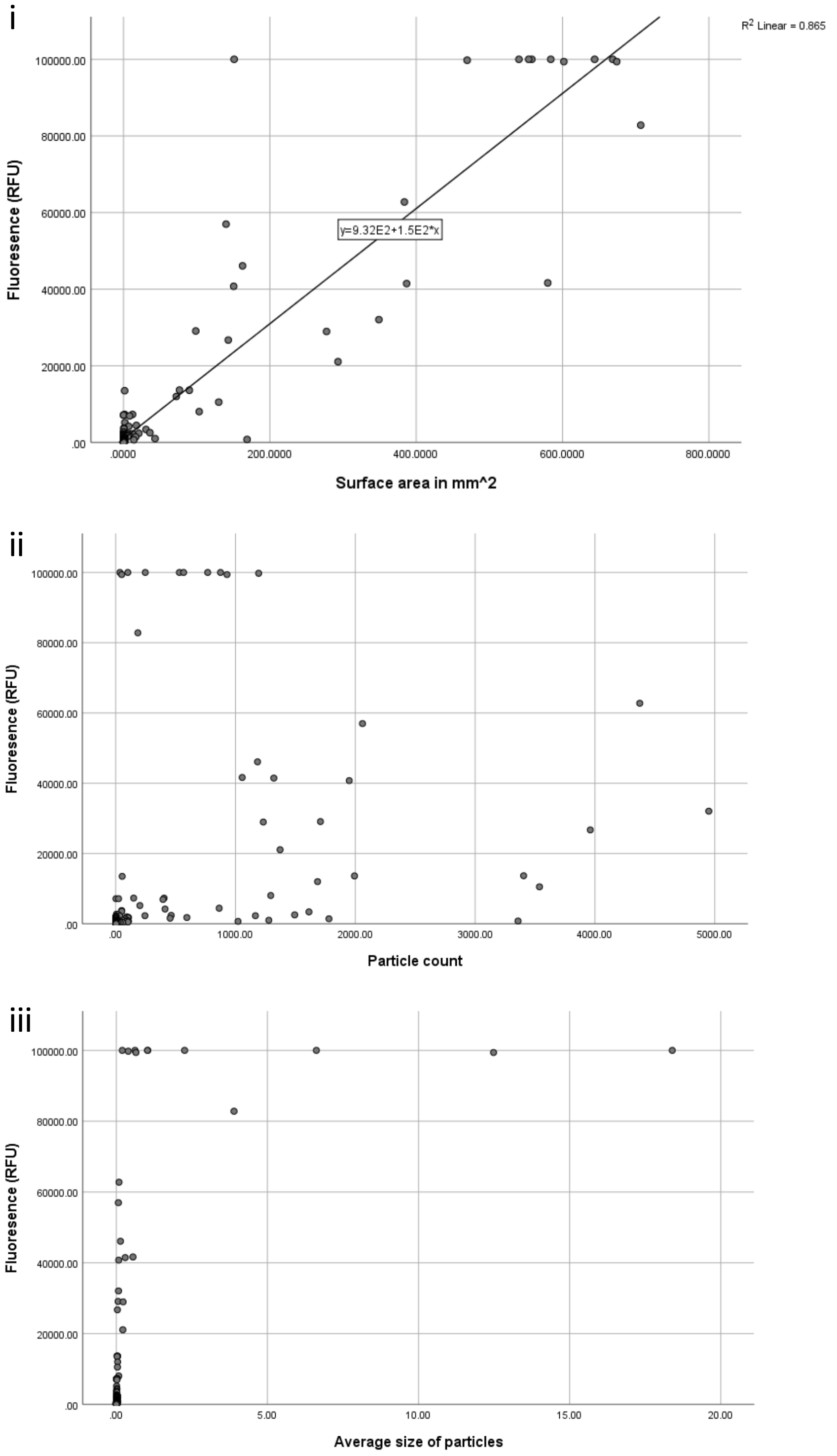

Supplementary Figure 1. Correlation of image analysis data with spectrofluorometric analysis data. (i) Total surface area very strongly correlated (r = 0.930, n = 244, p < 0.001), linear line of best fit shown; (ii) particle count weakly correlated (r = 0.344, n = 244, p < 0.001); (iii) Particle size moderately correlated (r = 0.555, n = 244, p < 0.001). RFU: relative fluorescence units.
