# Supplementary material for: Evaluating aerosol and splatter following dental procedures: Addressing new challenges for oral health care and rehabilitation
Source: J Oral Rehabil. 2020 Oct 8;48(1):61–72. doi: 10.1111/joor.13098 (PMC7537197; doi:10.1111/joor.13098)
Supplement: Supplementary file 2 — Fig S2 [file JOOR-48-61-s001.pdf]

Evaluating aerosol and splatter following dental procedures: addressing new challenges for oral healthcare and rehabilitation

Allison JR, Currie CC, Edwards DC, Bowes C, Coulter J, Pickering K, Kozhevnikova E, Durham J, Nile CJ, Jakubovics N, Rostami N, Holliday R

Supplementary Figure 2

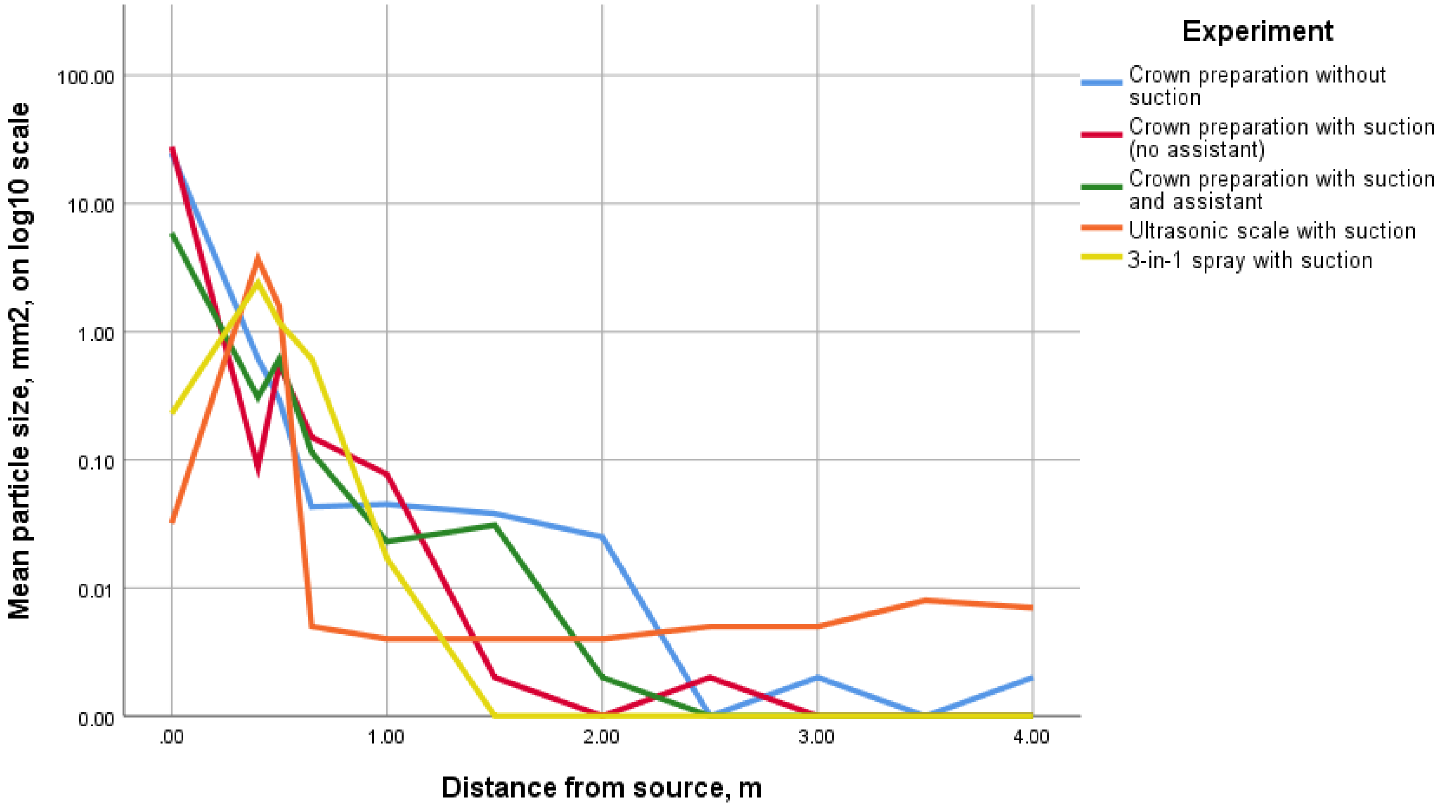

Supplementary Figure 2. Mean particle size (mm^2) per experimental condition presented by distance from source. Experimental rig (0, 0.5, 1, 1.5, 2, 2.5, 3, 3.5, 4 m) and mannequin (0.4 and 0.65 m) samples were included. Data presented on a logarithmic (base 10) scale. For each data point, the maximum value recorded from the three repetitions of each clinical procedure was used.
